# Cardiac manifestations of MIS-C: cardiac magnetic resonance and speckle-tracking data

**DOI:** 10.3389/fcvm.2023.1288176

**Published:** 2023-11-06

**Authors:** Lorenzo Scarduelli, Jean-Marie De Guillebon De Resnes, Dorothée Ducreux, Julie Bernardor, Mickael Afanetti, Audrey Dupont, Sébastien Barthelemy, Emmanuelle Gondon, Julien Leporati, Lisa Giovannini-Chami, Pamela Moceri

**Affiliations:** ^1^ Service de Pédiatrie, Hôpitaux Pédiatriques de Nice, CHU Lenval, Nice, France; ^2^ UR2CA, Faculté de Médecine, Equipe CARRES, Université Côte d’Azur, Nice, France; ^3^ Service de Radiologie, Centre Hospitalier Universitaire de Nice, Nice, France; ^4^ Faculté de Médecine, Université Côte d’Azur, Nice, France; ^5^ Service de Cardiologie, Centre Hospitalier Universitaire de Nice, Nice, France

**Keywords:** COVID-19, multisystem inflammatory syndrome in children (MIS-C), myocarditis, cardiac magnetic resonance, speckle-tracking imaging, cardiac injury

## Abstract

**Background:**

Cardiac involvement is central in MIS-C and represents the main cause of morbidity. In this study, we aimed to assess myocardial damage in patients with MIS-C using cardiac magnetic resonance (CMR) during the acute phase, as well as left ventricular and atrial longitudinal strain on admission, at discharge, and after 3 months.

**Methods:**

We performed a single-center prospective cohort study and case–control study. Between September 2020 and February 2022, we enrolled 39 patients hospitalized for MIS-C at our center. We performed left ventricular and atrial longitudinal 2D strain analysis on admission and during follow-up; echocardiographic data were compared to a matched control population. Patients above 4 years old with increased troponin underwent CMR.

**Results:**

Of 24 patients (mean age: 8.2 ± 4.9 years) who underwent CMR, 14 (58%) presented myocardial edema and 6 (25%) late gadolinium enhancement (LGE). LGE was associated with older age (*p* < 0.01), increased BMI (*p* = 0.03), increased ferritin levels (*p* < 0.001), lower left ventricular (LV) ejection fraction (*p* < 0.001), LV longitudinal strain (*p* = 0.004), left atrial (LA) strain (*p* = 0.05), and prolonged hospital stay (*p* = 0.02). On admission, LV ejection fraction, LV longitudinal strain, and LA strain were impaired, but each improved gradually over time; LVEF was the fastest to recover, while global LV longitudinal strain was still impaired as compared to controls after 3 months (*p* = 0.01).

**Conclusion:**

Our study demonstrates that myocardial injury is present in a quarter of MIS-C patients, and impaired LA and LV myocardial deformation persist for at least several weeks after the acute phase. CMR and LV/LA strain could help us to individualize follow-up of MIS-C patients.

## Introduction

Multisystem inflammatory syndrome in children (MIS-C) is a rare but severe complication associated with SARS-CoV-2 infection. It has been reported since April 2020 and it occurs in approximately 0.03% of young people (<21 years old) with SARS-COV2 infection confirmed by nasopharyngeal RT-PCR or by antibody testing (1). It is characterized by a generalized hyperinflammatory response (2), involving the heart in 90% of cases (3–5). Cytokine storm has been observed in MIS-C with a delayed interferon response and slow viral clearance (6). Initial descriptions have pointed out clinical similarities with Kawasaki disease and toxic shock syndrome (7).

Cardiac injury is frequent in children with MIS-C; however, the cardiac phenotype varies hugely among our patients. These patients may require intensive care and inotropic support for several days. Furthermore, cardiac magnetic resonance (CMR) data characterizing the myocardial damage that occurs during the acute phase of MIS-C are lacking. The identification of myocarditis is of crucial importance given the related outcomes (although these are largely dependent on the etiology): approximately one-quarter of patients may present with persistent cardiac dysfunction on follow-up, and 12%–25% of patients may deteriorate and either progress to end-stage heart failure with the need for heart transplant or die. The presence or absence of myocardial injury also determines the restrictions on physical activity and specific cardiovascular follow-up after the acute phase of MIS-C. Thus, it is of critical importance to assess MIS-C patients for the presence or absence of tissue-characterized myocarditis.

On this basis, we aimed to describe the acute and mid-term clinical, CMR, and echocardiographic abnormalities observed in our MIS-C patient cohort.

## Materials and methods

### Study design and patients

We conducted a prospective observational case–control study including all children with MIS-C hospitalized at our tertiary hospital center (Hôpital pédiatrique universitaire CHU-Lenval) between September 2020 and February 2022. MIS-C was defined according to the CDC/WHO case definition (8, 9), as follows:

- An individual aged <21 years presenting with fever ≥38.0 C and evidence of clinically severe illness requiring hospitalization, with multisystem (>2) organ involvement (cardiac, renal, respiratory, hematologic, gastrointestinal, dermatologic, or neurological) including severe cardiac illness (myocarditis, pericarditis, coronary artery dilatation, new onset left ventricular dysfunction, 2nd or 3rd degree atrio-ventricular block, or ventricular tachycardia) or rash and non-purulent conjunctivitis; and
- No alternative plausible diagnoses; and
- Laboratory evidence of inflammation and SARS-CoV-2 infection [based on reverse-transcriptase polymerase chain reaction (RT-PCR), serology, or antigen test].

Healthy controls were also enrolled to help determine normal values of 2D global longitudinal LV strain and LA strain. Healthy volunteers were recruited from the outpatient clinic to serve as controls; they were included if they had normal trans-thoracic echocardiography and were in sinus rhythm (children referred for functional heart murmur). They were matched to the study population on age and sex.

This study was conducted according to the guidelines of the Declaration of Helsinki and was approved by the local research ethics committee. After the provision of written information, guardians' consent was obtained for each participant.

### Data collection

Detailed demographic, clinical, and biological information was obtained from the participants' medical records and recorded in an anonymous database. Echocardiographic and CMR data were interpreted by two cardiologists and a radiologist specializing in cardiovascular disease.

### Echocardiographic data

**Figure 1:**
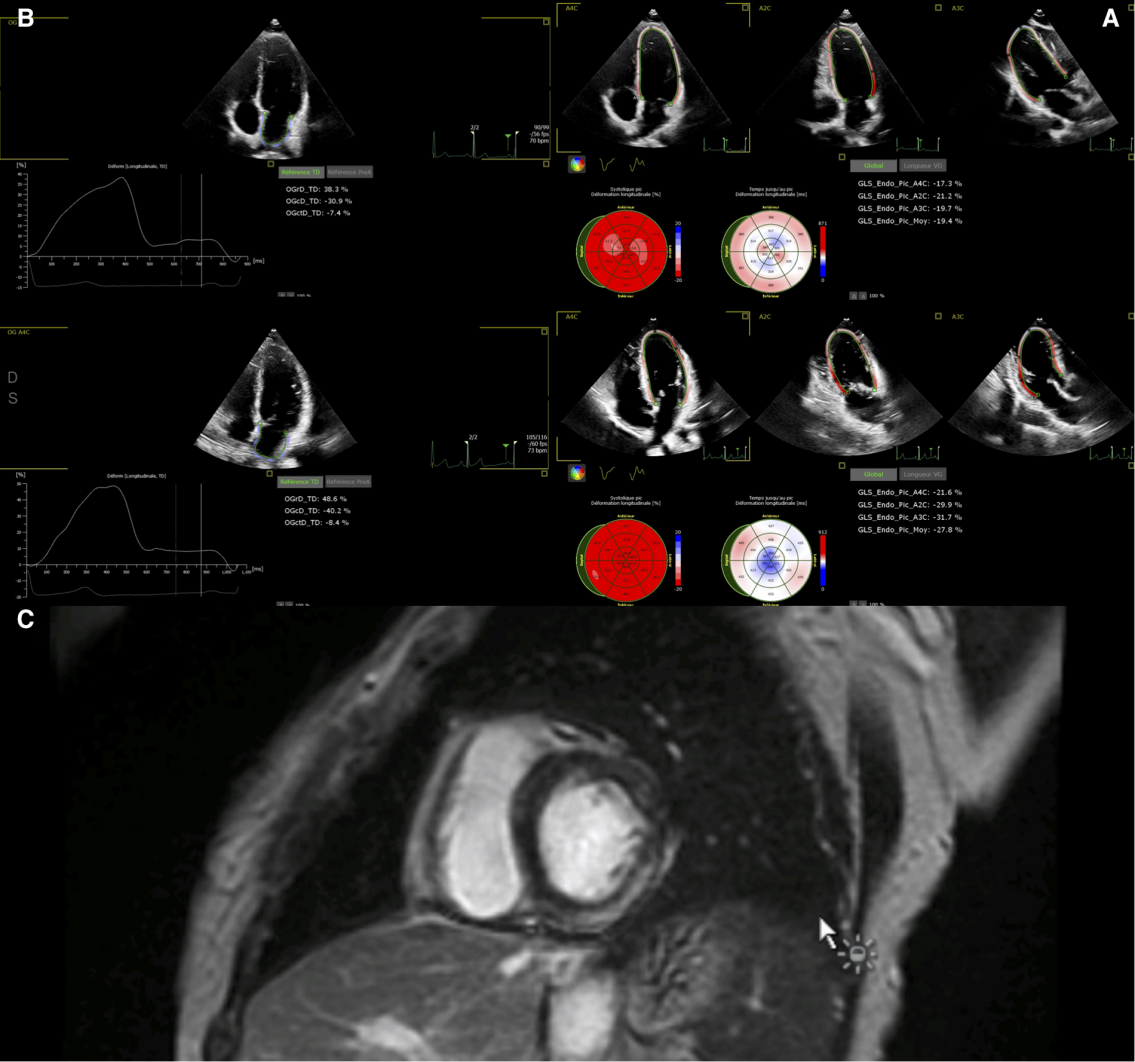
(**A**) Global left ventricular longitudinal strain in a 12-year-old boy before discharge (upper panel) and after 6 months. (**B**) Left atrial strain in the same 12-year-old boy before discharge (upper panel) and after 6 months. (**C**) Cardiac MRI in a 7-year-old boy 3 days after admission, showing late gadolinium enhancement within the inferior and lateral wall.

Echocardiography was performed using a Philips Affiniti 70 or EPIQ 7 ultrasound machine (Philips Medical Systems, Eindhoven, Netherlands) with an S9-2 or X5-1 probe. 2D imaging included the assessment of left ventricular (LV) segmental and global function: LV ejection fraction (EF), assessed using the Simpson method; presence or absence of segmental LV wall abnormalities; mitral regurgitation; right ventricular dysfunction; pericardial effusion; coronary artery dilatation; and perivascular brightness of the coronary arteries. Left ventricular strain was assessed using speckle-tracking echocardiography of the apical long axis, obtained from the apical four-, two- and three-chamber views, with a frame rate of 60–80/s. The analysis was either performed directly on the ultrasound machine or performed offline using commercially available semi-automated 2D strain software (QLAB, Philips Medical System) (Figure 1A). Left atrial strain was assessed using the apical 4-chamber view (QLAB, Philips Medical System) (Figure 1B). We recorded the peak longitudinal left atrial strain (reservoir strain).

### CMR data

Patient with a significant increase in troponin (or if their LV function was impaired and failed to improve after medical therapy) underwent CMR study.

Studies were performed on a General Electric Optima MR450 1.5 T CMR scanner. We used Lake Louise criteria (10) to determine the presence or absence of myocarditis. The acquisition protocol included conventional balanced steady state free precession cine images in ventricular 2-, 3-, and 4-chamber planes. Following these acquisitions, 0.1 mmol/kg of a gadolinium contrast agent (Dotarem) was administered and flushed with isotonic saline serum. LGE images in three long-axis and a stack of short-axis imaging planes were obtained with a breath-hold phase-sensitive inversion recovery sequence 5–15 min after the contrast injection. The presence and location of LGE were assessed visually (Figure 1C). End-diastolic and end-systolic endocardial borders were drawn semi-automatically for LV volumes and LVEF assessment. The imaging protocol included cine, STIR, and LGE imaging at standardized apical, mid-cavity, and basal short-axis levels. T1 mapping, T2 mapping, and extracellular volume are not available at our center. For purposes of simplicity, patients were classified according to the presence or absence of LGE or myocardial edema on CMR.

### Statistical analysis

Data are summarized in the form mean ± standard deviation for continuous variables distributed normally, median [95% confidence interval] for other continuous variables, and number of subjects (%) for categorical variables. Comparisons between patients with and without LGE were performed using the Student's *t*-test for normally distributed variables or using the Wilcoxon test otherwise. Categorical variables were compared using Fisher's exact test. These analyses were performed using R++ version 1.5.08 (Paris, France). In all analyses, statistical significance was defined as a *p*-value <0.05.

## Results

### Demographics and clinical presentation

Between September 2020 and February 2022, we enrolled 39 patients with MIS-C. Patient characteristics are presented in Table 1. Our population consisted of a majority of boys (31, 79%); the mean age was 8.2 ± 4.9 years. Most patients had previously been healthy, with the exception of one girl with a history of junctional tachycardia and one boy with Brugada pattern on ECG (which was discovered during the episode of fever related to MIS-C).

**Table 1:** General characteristics of the study population.

* *	*n* = 39
Male, *n* (%)	31 (79)
Age at symptom onset, years	8.2 [3.3–13.1]
Duration between fever and hospitalization, days	4 [2.5–5.9]
Gastrointestinal symptoms, *n* (%)	25 (64)
Cutaneous symptoms, *n* (%)	21 (54)
Lymphadenopathy, *n* (%)	7 (18)
Headaches or neck pain, *n* (%)	7 (18)
Conjunctivitis, *n* (%)	6 (15)
Chest pain or difficulty breathing, *n* (%)	4 (10)
Swollen hands and feet, *n* (%)	3 (8)
COVID serology positive, *n* (%)	31 (79)
COVID PCR positive, *n* (%)	7 (18)
CRP level, mg/L	230 (190–267)
Leucocytes, /L	17.7 ± 7.4 × 10^9^
Ferritin, µg/L	352.5 [290–599]

All patients presented with persistent fever (median duration before admission: 4 days [2.5–5.9]). Twenty-five patients (64%) had gastrointestinal symptoms (diarrhea, vomiting, or abdominal pain) and 21 (54%) had cutaneous (skin rash) or mucous membrane involvement (red or swollen lips). Less frequently, patients presented with lymphadenopathy (*n* = 7, 18%); seven patients (18%) suffered from either headaches or neck pain; six (15%) had conjunctivitis; four (10%) presented with chest pain or difficulty breathing; and three (8%) had swollen hands and feet.

All patients presented a marked inflammatory state: their median CRP level was 230 [190–267] mg/L, mean number of leukocytes was 17.7 ± 7.4 × 10^9^/L, and median ferritin level was 352.5 [290–599] µg/L. Previous SARS-CoV-2 infection was confirmed by positive RT-PCR in 20.5% of patients (*n* = 8) and by positive IgG antibody against SARS-CoV-2 in 79% (*n* = 31).

### Cardiovascular presentation

Nine patients (23%) had a pathologic electrocardiogram (ECG) during the course of their hospital stay: eight presented a first-degree atrio-ventricular block (the PR interval was monitored during the hospital stay, and prolongation appeared after the 3rd day of evolution in five patients), whereas two patients (5%) presented with ST segment abnormalities. No sustained arrythmia was observed.

Three patients (8%) did not present any cardiac involvement; eight (21%) had sub-clinical cardiac injury; and six patients (15%) were admitted with Kawasaki-like presentation (either coronary dilatation or coronary perivascular brightness). Eight patients (21%) presented with clinical pericarditis, whereas seven (18%) had evidence of severe heart failure. “Kawasaki-like” patients were younger than others (4.4 [1.5–8.4] vs. 9.1 [7.6–13.2] years old; *p* = 0.03).

Cardiac biomarkers (BNP and/or troponin) were elevated in 31 patients (79%). Median BNP measurement was 814 [666–1,306] pg/ml, whereas median troponin level was 500 [341–1,833] ng/L. Maximum troponin level was 8,752 ng/L, while maximum BNP dosage was 4,145 pg/ml. In 10 patients, BNP was elevated but normal troponin levels were observed, while only one patient had increased troponin but normal BNP.

### Echocardiographic findings on admission

Echocardiographic findings on admission, at discharge, and at 3-month follow-up are presented in Table 2.

**Table 2:** Echocardiographic characteristics of MIS-C patients on admission, at discharge, and at 3-month follow-up.

* *	Admission	Discharge	3 months	*p* *A* vs. *D*	*p* *A* vs. M3
*E*/*A* ratio	1.7 [1.4–2.0]	1.7 [1.6–2.4]	1.6 [1.4–1.7]	0.37	0.26
*E* wave, cm/s	101.5 ± 20.1	101.5 ± 16.2	93.7 ± 13.9	0.59	**0** **.** **02**
*E*/*E*′ ratio	7.16 ± 1.8	7.42 ± 1.7	5.6 ± 1.1	0.52	**<0**.**0001**
*E*′ wave, cm/s	14.7 ± 3.2	14.1 ± 3.2	17.5 ± 2.6	0.64	**0**.**0001**
TAPSE, mm	17.1 ± 4.0	27 ± 1.4	22.3 ± 2.2	0.11	0.30
LVEF, %	51.7 ± 12.1	60.3 ± 6.6	63.2 ± 5.4	**<0**.**0001**	**<0**.**0001**
Global LV longitudinal strain, %	−14.4 ± 4.3	−18.2 ± 3.4	−19.8 ± 3.6	**<0**.**0001**	**<0**.**0001**
Peak LA strain, %	22.9 ± 9.2	28.8 ± 9.1	36.2 ± 10.2	**0**.**0037**	**0**.**0007**

*A*, admission; *D*, discharge; LA, left atrial; LV, left ventricular; LVEF, left ventricular ejection fraction; M3, 3 months after hospitalization; TAPSE, tricuspid annular plane systolic excursion.

Bold values indicate significant *p*-values < 0.05.

Mean LV ejection fraction on admission was 49 ± 11.8%: seven patients (18%) presented with severe LV dysfunction (LVEF ≤ 35%), and 14 (36%) with moderately impaired LV function (LVEF between 36% and 54%). Ten patients presented with evidence of segmental LV wall motion abnormality, especially within the anterior and septal wall. One patient had severe biventricular dysfunction. LV ejection fraction significantly improved at discharge (*p* < 0.001) and at 3-month follow-up (*p* < 0.001), while E/E' ratio decreased slightly at 3-month follow-up. Seventeen patients (44%) had evidence of significant but mild mitral regurgitation on admission, and nine patients (23%) had pericardial effusion. Seven patients (18%) showed evidence of coronary involvement (either dilatation or perivascular brightness of the coronary arteries).

Mean global longitudinal LV strain improved significantly from admission (−14.4 ± 4.3%) to discharge (−18.2 ± 3.4%; *p* < 0.001) and 3-month follow-up (−19.8 ± 3.6; *p* < 0.001). Mean LA peak strain on admission was also improved at discharge and after 3 months (*p* < 0.001). As compared to controls (Table 3), global longitudinal LV strain was significantly impaired in MIS-C patients on admission and at discharge, and also at 3-month follow-up (*p* < 0.001, *p* < 0.001, and *p* = 0.01, respectively). Left atrial reservoir strain was impaired in MIS-C patients on admission (*p* < 0.001) and at discharge (*p* = 0.02), but returned to normal levels at 3-month follow-up (*p* = 0.82).

**Table 3:** Comparison of speckle-tracking data between MIS-C patients and controls.

** **	Controls	*p* *A* vs. controls	*p* *D* vs. controls	*p* M3 vs. controls
Global LV longitudinal strain, %	−22.0 ± 2.3	**<0**.**0001**	**<0**.**0001**	**0**.**01**
Peak LA strain, %	35.5 ± 9.3	**<0**.**0001**	**0**.**024**	0.82

*A*, admission; *D*, discharge; LA, left atrial; LV, left ventricular; M3, 3 months after hospitalization.

Bold values indicate significant *p*-values < 0.05.

### CMR findings

CMR was performed in 24 patients after a median delay of 6 days [4.3–10.3] after onset of symptoms. Myocardial edema was found in 14 patients (58%). LGE was identified in six patients (25%), among whom five patients presented with severe heart failure (71% of severe heart failure patients). LGE+ was more frequently observed in patients with heart failure.

Patients with LGE were older (LGE-: 8.9 [7.3–13.5] vs. LGE+: 14.1 [9.9–15.7]; *p* = 0.0003) and had higher body mass index (*p* = 0.03) as compared to patients without LGE. Patients with LGE also had lower LVEF (*p* = 0.004), decreased global LV longitudinal strain (*p* = 0.004), and left atrial reservoir strain (*p* = 0.05) (Figure 1).

**Figure 2:**
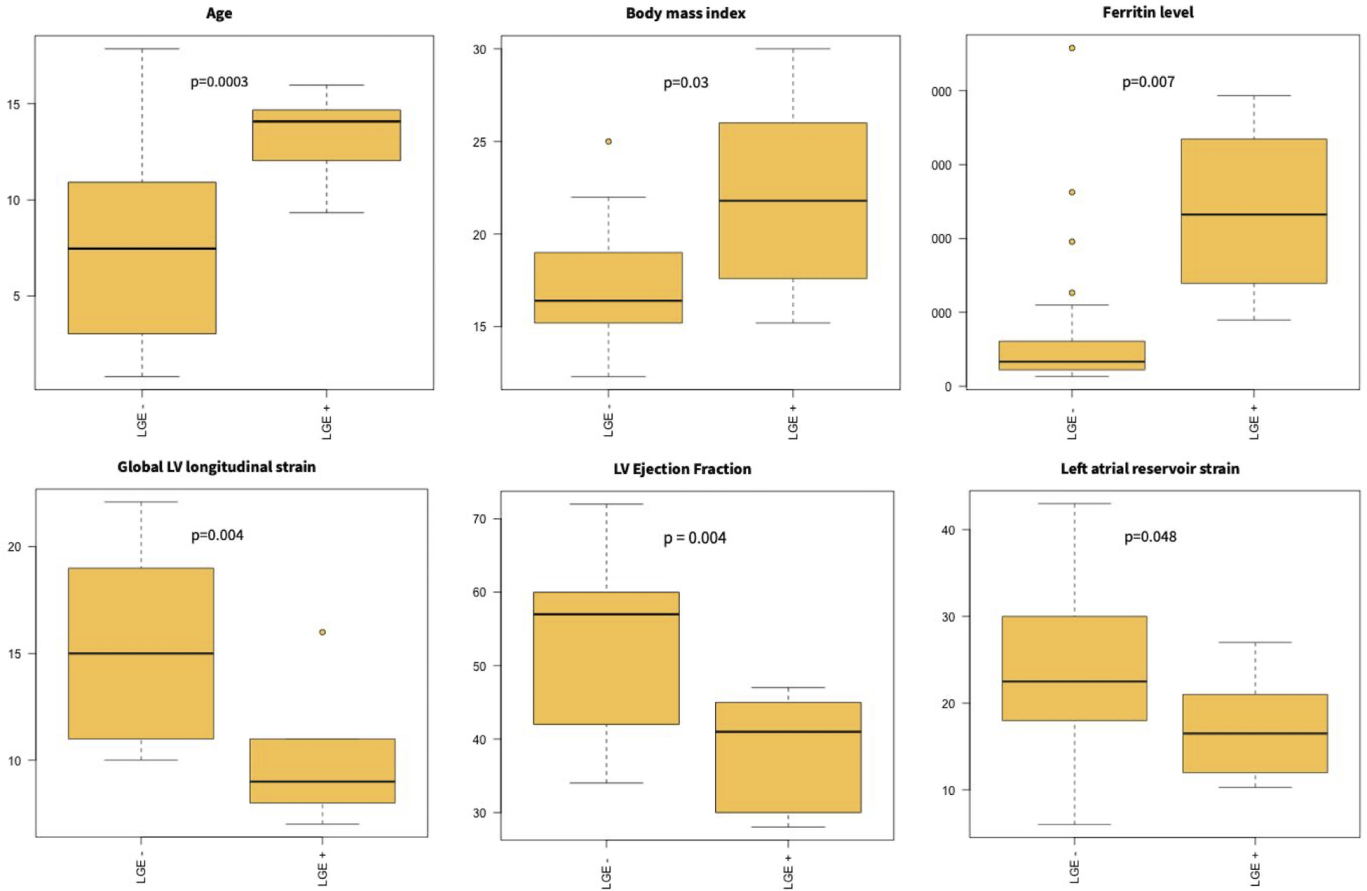
Myocardial injury in MIS-C. Patients with LGE were older and had higher BMI. They had higher ferritin levels and lower left ventricular ejection fraction and longitudinal strain on admission.

There was no difference in peak CRP (*p* = 0.23) or leukocyte count (*p* = 0.94) according to the presence of LGE, but higher levels of ferritin (LGE-: 534.0 [315.2–1,000.0] vs. LGE+: 2,323.0 [1,394.5–3,345.5]; *p* = 0.007) were observed in patients with LGE. No difference in BNP or troponin was observed (for BNP, LGE-: 834.5 [427.9–1,472.5] vs. LGE+: 1,185.5 [442.9–2,027.1], *p* = 0.41; for troponin, LGE-: 109.0 [69.2–1,200.3] vs. LGE+: 1,495.0 [170.4-7,503.2], *p* = 0.10) (Figure 2). Patients with LGE were more likely to have a prolonged hospital stay (*p* = 0.02).

### Clinical evolution

The most common treatments administered during the acute phase included aspirin in 38 patients (97.4%), low-molecular-weight heparin anticoagulation in 10 (25.6%), intravenous immunoglobulin in 38 (97.4%), intravenous corticosteroids in 36 (92.3%), and anakinra in 1 (2.6%). Eighteen patients (46%) required PICU admission, 14 of these (36%) requiring vasopressor or inotrope support: 5 patients (13%) required norepinephrine infusion, and dobutamine was required in 14 (36%).

All patients had recovered normal LVEF upon discharge and all survived after a median follow-up period of 7.5 months [4.5–9.7]. Three months after discharge from hospital, MIS-C patients did not differ significantly from controls in terms of LA strain, but global LV longitudinal strain was still impaired (*p* = 0.01).

Patients with LGE on initial CMR underwent an exercise test and a control CMR 6 months after MIS-C. All six of these patients had a normal control CMR with no residual LGE or myocardial edema, as well as a normal exercise test.

## Discussion

Our study demonstrates the presence of tissue-characterized myocarditis with LGE in one-quarter of our MIS-C patient cohort who underwent CMR within the first month after hospitalization. LGE occurred more frequently in older children and was associated with higher BMI, higher levels of ferritin, lower LVEF, lower LV global longitudinal, and LA strain. Myocardial injury on CMR was associated with a prolonged hospital stay. Three months after discharge, LV global longitudinal strain was still impaired as compared to a control population, while other echocardiographic parameters had normalized.

Our findings are supported by recent studies (11, 12) showing evidence of CMR myocarditis in approximately 16%–35% of MIS-C patients. Studies investigating the presence of LGE during the acute phase of MIS-C are scarce, and preliminary studies have not identified LGE in MIS-C patients (13, 14). LGE has probably been underestimated in those studies as a result of the delay between hospitalization and MRI (2–3 months), as compared to the CARDOVID cohort (11), where the median delay for CMR was 28 days, and our study (6 days). LGE findings on CMR reflected myocardial injury. As LGE was not related to LV ejection fraction, myocardial injury could be found even in the presence of preserved or mildly reduced LV systolic function. LGE can suggest active inflammation but is less effective in detecting “borderline myocarditis” (15). This underlines the importance of CMR within a reasonable time delay in order to detect LGE, as the extent of LGE will decrease over time, explaining the number of false negatives obtained when performing an MRI late after the onset of MIS-C. Indeed, children with LGE should probably undergo strict cardiovascular follow-up given the potential risk of myocardial scarring and fibrosis, which might lead to arrythmia. Residual myocardial damage 6-9 months after MIS-C, as assessed by CMR, appears to be minimal according to a recent study (16), supporting the role of early MRI in detecting high-risk patients. In contrast, the American College of Rheumatology Clinical Guidance for MIS-C recommends cardiac MRI 2 to 6 months after MIS-C diagnosis in patients with LV ejection fraction <50% during the acute phase of illness (17). Our data and previous studies suggest that LGE occurs earlier than 2 months and is not related to LV systolic dysfunction. CMR is helpful to determine the presence and extent of myocardial inflammation, suggested typically by LGE. In the absence of LGE, after complete recovery from MIS-C, patients should not be advised against exercise, especially given that the prevalence of overweight in MIS-C is 25% (7).

Given the overall excellent cardiovascular outcome of MIS-C, the presence of LGE on CMR could help us detect children with a higher risk of cardiovascular complications. This should also be borne in mind when considering the resumption of sporting activity in school-aged patients. Indeed, myocarditis is a risk factor for sudden cardiac death in athletes (18–20). Both the European Society of Cardiology (21) and the American Heart Association (22) recommend abstinence from moderate- to high-intensity exercise for a period of 3–6 months after myocarditis. The timing for return to sport could be guided by CMR LGE, underlining its importance in detecting active inflammation.

The cardiovascular presentation of MIS-C varies widely from sub-clinical cardiac injury to myocarditis with severe heart failure. We report here that MIS-C with coronary artery dilatation or perivascular brightness is related to younger age at presentation. This supports low-dose aspirin treatment for at least 6 weeks (23) in young patients with Kawasaki-like presentation, as well as appropriate follow-up using coronary *Z*-scores. Kawasaki syndrome and MIS-C might belong to the common spectrum of pathogen-triggered hyperinflammatory state and share a common presentation in young children, whereas older children mostly have a clinical presentation involving heart failure, myocarditis, and gastrointestinal symptoms. On another hand, overweight has been found to be associated with the occurrence of MIS-C (7, 24), and in our study, BMI was related to the presence of LGE on MRI in MIS-C. This might explain the potentially poorer prognosis of MIS-C in obese children (25, 26).

Our study confirms the overall excellent prognosis of MIS-C. All patients were discharged alive, and LV ejection fraction had recovered after 3 months in all cases, even following severe cardiogenic shock. However, although we observed improvement in LV and LA strain over time, global LV longitudinal strain was still impaired 3 months post-discharge as compared to controls, suggesting subclinical myocardial dysfunction. We thus confirm that global LV longitudinal strain is last to normalize in MIS-C (12). The decrease in LA strain, although this improves over time, might suggest the presence of subtle diastolic dysfunction and may prompt prolonged follow-up in those children, especially in those with risk factors such as obesity.

### Limitations

This was a single-center study, thus limiting the scope for extrapolation from our data. However, the early CMR scanning conducted in our patients increases the robustness of our data. Although LGE provides information on myocardial inflammation and necrosis status, this does not offer any clues regarding the pathogenesis of MIS-C. Our data were collected mostly during the outbreaks of the alpha, delta, and omicron variants of COVID. Whether MIS-C related to future variants will lead to the same conclusions remains unknown.

## Conclusion

MIS-C physiopathology remains unclear. The possible presence of myocarditis with myocardial injury prompts us to make the generalization that early CMR scanning should be performed in MIS-C patients whenever possible. Standard and speckle-tracking echocardiography are especially useful for diagnosis but also for follow-up. Stratifying cardiac risk in MIS-C patients using CMR and echocardiography will help us to individualize follow-up and probably avoid potential cardiac complications in this young population.

## Data Availability

The raw data supporting the conclusions of this article will be made available by the authors, without undue reservation.
